# Interactive effects of elevated CO_2_ and precipitation change on leaf nitrogen of dominant *Stipa* L. species

**DOI:** 10.1002/ece3.1581

**Published:** 2015-07-03

**Authors:** Yaohui Shi, Guangsheng Zhou, Yanling Jiang, Hui Wang, Zhenzhu Xu, Jian Song

**Affiliations:** 1 State Key Laboratory of Vegetation and Environmental Change, Institute of Botany, Chinese Academy of SciencesBeijing, 100093, China; 2 University of Chinese Academy of SciencesBeijing, 100049, China; 3 Chinese Academy of Meteorological SciencesBeijing, 100081, China

**Keywords:** Elevated CO_2_, interactive effect, leaf N concentration, precipitation change, *Stipa* L.

## Abstract

Nitrogen (N) serves as an important mineral element affecting plant productivity and nutritional quality. However, few studies have addressed the interactive effects of elevated CO_2_ and precipitation change on leaf N of dominant grassland genera such as *Stipa* L. This has restricted our understanding of the responses of grassland to climate change. We simulated the interactive effects of elevated CO_2_ concentration and varied precipitation on leaf N concentration (N_mass_) of four *Stipa* species (*Stipa baicalensis*, *Stipa bungeana*, *Stipa grandis,* and *Stipa breviflora*; the most dominant species in arid and semiarid grassland) using open-top chambers (OTCs). The relationship between the N_mass_ of these four *Stipa* species and precipitation well fits a logarithmic function. The sensitivity of these four species to precipitation change was ranked as follows: *S. bungeana *> *S. breviflora *>* S. baicalensis *> *S. grandis*. The N_mass_ of *S. bungeana* was the most sensitive to precipitation change, while *S. grandis* was the least sensitive among these *Stipa* species. Elevated CO_2_ exacerbated the effect of precipitation on N_mass_. N_mass_ decreased under elevated CO_2_ due to growth dilution and a direct negative effect on N assimilation. Elevated CO_2_ reduced N_mass_ only in a certain precipitation range for *S. baicalensis* (163–343 mm), *S. bungeana* (164–355 mm), *S. grandis* (148–286 mm), and *S. breviflora* (130–316 mm); severe drought or excessive rainfall would be expected to result in a reduced impact of elevated CO_2_. Elevated CO_2_ affected the N_mass_ of *S. grandis* only in a narrow precipitation range. The effect of elevated CO_2_ reached a maximum when the amount of precipitation was 253, 260, 217, and 222 mm for *S. baicalensis*, *S. bungeana*, *S. grandis,* and *S. breviflora*, respectively. The N_mass_ of *S. grandis* was the least sensitive to elevated CO_2_. The N_mass_ of *S. breviflora* was more sensitive to elevated CO_2_ under a drought condition compared with the other *Stipa* species.

## Introduction

The atmospheric CO_2_ concentration has been rising from preindustrial values of approximately 280–390 ppm at present and is expected to reach approximately 450 and 560 ppm under low (RCP2.6) and medium (RCP4.5) scenarios, respectively, in the 21st century (IPCC, 2013). Accompanied with an increase in greenhouse gases, many midlatitude arid and semiarid regions will likely experience less precipitation, and more extreme weather events may arise (IPCC, 2013). Elevated atmospheric CO_2_ concentration and simultaneous precipitation change directly or indirectly affect plant physiology and growth (Reich et al. 2001; Xu and Zhou 2006; Sun et al. 2009; Ghannoum et al. 2010; Albert et al. 2011; Tian et al. 2013). Grassland is an important part of the terrestrial ecosystem and plays a significant role in the functioning and structure of the Earth’s ecosystems; grasslands are generally thought to be very vulnerable and sensitive to climate change (Weltzin et al. 2003; Ji et al. 2005; Zhang et al. 2007). Leaf nitrogen (N) is closely related to photosynthesis, and leaf N concentration is also one of the key traits of the economic spectrum of leaves (Wright et al. 2004; Feng et al. 2009). The leaf N concentration (N_mass_) of a plant is determined by both genetic characteristics and environmental factors (precipitation, temperature, CO_2_ and O_3_), and reflects the ability of a plant to adapt to the environment. Many studies have addressed the effects of elevated atmospheric CO_2_ concentrations or precipitation change on plant N_mass_. These studies have shown that elevated atmospheric CO_2_ concentrations can result in a decrease in N_mass_, while drought stress can increase N_mass_ (Ainsworth and Long 2005; Teng et al. 2006; Bloom et al. 2010; Lee et al. 2011; Zhou et al. 2011; Housman et al. 2012). However, some researchers found that elevated CO_2_ did not affect N_mass_ (Watling et al. 2000; Novriyanti et al. 2012; Li et al. 2013), and others indicated that N_mass_ decreased with decreasing rainfall (Xu and Zhou 2006; Galmés et al. 2007), possibly depending on plant species.

The responses of plant growth and physiology to climatic change, in a multifactor context, may not be predictable from a single factor experiment. However, most experiments have focused on the effects of an individual factor; therefore, multifactorial experiments are urgently needed to reveal the integrated responses of plants to environmental changes (Albert et al. 2011; Vile et al. 2012; Hou et al. 2013; Xu et al. 2014). Grassland dominated by *Stipa*, a group of species with good palatability and high forage value, is widespread in North China as the part of the Euro-Asia steppe, an ecosystem that has experienced severe degradation during recent decades (Bai et al. 2004; Zhang et al. 2007; Xu et al. 2014). Previous studies were mainly concerned about the effect of precipitation change; the interaction with CO_2_ concentration was unclear. The increase in the CO_2_ concentration and changes in precipitation will occur simultaneously in the future (IPCC, 2013), and the responses of *Stipa* to changing precipitation may vary in an environment with a higher CO_2_ concentration. Leaf N_mass_ affects the decomposition rate of plant litter and is closely related to forage quality (Gorissen and Cotrufo 2000; Vitousek et al. 2002; Pleijel and Uddling 2012). In this study, open-top chambers (OTCs) were used to (1) investigate the interactive effects of elevated CO_2_ and precipitation change on N_mass_; (2) quantify the relationship between N_mass_ and precipitation; and (3) elucidate the mechanisms involved the N_mass_ response to elevated CO_2_ and precipitation change.

## Materials and Methods

### Plant materials and experimental design

Four *Stipa* species (*Stipa baicalensis*, *Stipa bungeana*, *Stipa grandis,* and *Stipa breviflora*), which are the most typical species in the arid and semiarid grassland of China, were chosen for this experiment. The experiment was conducted at the Institute of Botany, Chinese Academy of Sciences, in 2011, using OTCs. *S. baicalensis*, *S. bungeana*, *S. grandis,* and *S. breviflora* seeds were collected from natural grasslands in Hulunber (49°190′N, 119°55′E), Ordos (39°29′N, 110°11′E), Xilinhot (44°08′′N, 117°05′′E), and Ulanqab (41˚43′’N, 111˚52′E) in the autumn of 2010. The seeds were sterilized in a 0.5% potassium permanganate solution for 8 min before sowing. The soil (N_mass_: 1.45 g·kg^−1^) had been collected from the original grassland in Xilinhot, Inner Mongolia, and was placed into plastic pots (0.56 L).

Three CO_2_ concentration treatments (ambient, 450 and 550 ppm) with three replications were tested in a total of nine OTCs. The hexagonal structure of the OTCs, which were fabricated using an aluminum frame lined with colorless transparent glass, had a length and height of 0.85 and 1.8 m, respectively. Pure CO_2_ gas was released through a PVC tube connected to an air-exhaust blower mounted at the base of the OTCs. The input of CO_2_ gas was automatically controlled, and an air sample from the middle of the chamber was drawn into a CO_2_ sensor (eSENSE-D, SenseAir, Delsbo, Sweden) to monitor the concentration change every minute. The natural precipitation of the seed provenances was similar for pairs of species, that is, (1) *S. baicalensis* and *S. bungeana* and (2) *S. grandis* and *S. breviflora*. To facilitate a comparison of the species pairs, the baseline precipitation (June, July, and August) data from Hulunber (240 mm) and Xilinhot (217 mm) were used for calculating the experimental precipitation rates. That is, two sets of five precipitation levels (−30%, −15%, control, +15%, and +30%) were used. These were based on the average monthly precipitation (June, July, and August) in different regions of the two pairs of species from 1978 to 2007. Every precipitation level had two replicates in each OTC. The monthly precipitation (mm) of each level (Table1) was converted into an irrigation amount (ml), and this was supplied every 3 days.

**Table 1:** Average monthly precipitation from 1978 to 2007 in the provenances of the four species

Species	Month	Precipitation (mm)				
		−30%	−15%	Control	+15%	+30%
*S. baicalensis*	June	36	44	51	59	67
*S. bungeana*
	July	62	75	88	101	114
	August	70	85	100	115	130
	Total	168	204	240	275	311
*S. grandis*	June	39	47	56	64	72
*S. breviflora*
	July	65	79	93	107	121
	August	47	57	68	78	88
	Total	151	183	217	249	281

After sowing on 18 April 2011, the seedlings were first cultured in a greenhouse (day/night temperature 26–28°C/18–20°C, maximum photosynthetic photon flux density of 1000 *μ*mol·m^−2^·s^−1^). Four healthy seedlings with a uniform growth pattern were retained in each pot when the fourth leaf appeared. A total of 360 pots (90 pots for each species) were randomly selected and moved into the OTCs (10 pots for each species in each chamber) on 23 May. Thus, there were six replicates (six pots, each with four plants) per treatment for each species. Before CO_2_ enrichment and irrigation started on 31 May, we weighed every pot with soil and plants to ensure that initial soil moisture was consistent. During the experiment, we monitored the CO_2_ supply system every day, watered at 16:00 every 3 days, and kept the glass walls clean.

### Sampling and analysis

After harvesting on 2 September 2011, the leaves were dried at 65°C to a constant weight and leaf biomass was measured using an electronic balance. The leaf N concentration (N_mass_) was determined using a Vario EL III elemental analyzer (Elementar Analysensysteme GmbH, Hanau, Germany). Total leaf N (N_total_) = leaf biomass × N_mass_. The relative effects of N_mass_ (*α*_Nmass_), leaf biomass (*α*_biomass_), and N_total_ (*α*_Ntotal_) can be expressed using the following equation: 

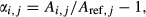
where *α*_*i,j*_ is the relative effect on variable *j* of treatment *i* in relation to the control, *A*_*i,j*_ is the value of variable *j* of treatment *i*, and *A*_ref*,j*_ is the value of variable *j* of the control. Controls were only used to calculate experimental effects; by definition, *α* is zero for all variables in the control (Pleijel and Uddling 2012).

### Statistical tests

All statistical analyses on the N_mass_ and N_total_ values were performed using SPSS 16.0 (SPSS Institute Incorporated, Chicago, IL, USA). The effects of elevated CO_2_ and precipitation change were analyzed using ANOVA (*P *= 0.05). Differences between the means of the elevated CO_2_ or precipitation changes were compared using Duncan’s multiple range test at a 0.05 probability level.

## Results and Analysis

### Responses of N_mass_ to elevated CO_2_ and precipitation changes

**Figure 1:**
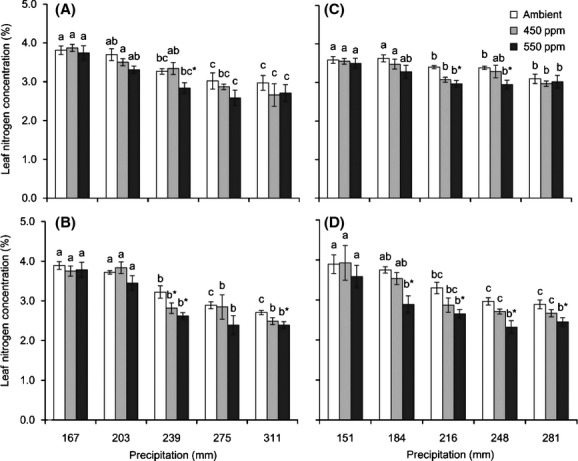
Interactive effects of changing precipitation and CO_2_ on the N_mass_ of four *Stipa* species: *S. baicalensis* (A), *S. bungeana* (B), *S. grandis* (C), and *S. breviflora* (D). Different lower case letters indicate significant differences among precipitation treatments for the same CO_2_ concentration (*P *< 0.05); * indicates significant differences between CO_2_ concentrations for the same level of precipitation (*P *< 0.05).

The relationship between N_mass_ and precipitation for the four *Stipa* species was better observed using a logarithmic function (Fig.1, Table2). The equations in Table2 showed a better linear relationship between *y* and ln*x* (*y*: N_mass_, *x*: precipitation). The slope (*a*) reflected the degree of influence of the precipitation change on N_mass_. A larger ¦*a*¦ indicated a greater effect of precipitation change on N_mass_. Under the same CO_2_ concentration conditions, the sensitivities of the N_mass_ of the four species to precipitation change were ranked as: *S. bungeana *> *S. breviflora > S. baicalensis > S. grandis*. The N_mass_ of *S. bungeana* was the most sensitive to precipitation change, while *S. grandis* was the least sensitive among these *Stipa* species. Compared with the ambient level, high CO_2_ concentration intensified the effect of precipitation change on N_mass_.

**Table 2:** Relationship between N_mass_ and precipitation under different CO_2_ concentrations

Species	CO_2_ concentration	Equation	*R* ^2^	*P*
*S. baicalensis*	Ambient	*y* = −1.526ln(*x*) + 11.677	0.6749	<0.01
	450 ppm	*y* = −1.963ln(*x*) + 13.954	0.7634	<0.01
	550 ppm	*y* = −1.853ln(*x*) + 13.144	0.6778	<0.01
*S. bungeana*	Ambient	*y* = −2.06ln(*x*) + 14.52	0.8892	<0.01
	450 ppm	*y* = −2.262ln(*x*) + 15.482	0.6861	<0.01
	550 ppm	*y* = −2.531ln(*x*) + 16.73	0.7836	<0.01
*S. grandis*	Ambient	*y* = −0.765ln(*x*) + 7.5102	0.5719	<0.01
	450 ppm	*y* = −0.877ln(*x*) + 7.9664	0.5036	<0.01
	550 ppm	*y* = −0.869ln(*x*) + 7.7904	0.4344	<0.01
*S. breviflora*	Ambient	*y* = −1.816ln(*x*) + 13.078	0.7649	<0.01
	450 ppm	*y* = −2.214ln(*x*) + 14.991	0.6602	<0.01
	550 ppm	*y* = −1.906ln(*x*) + 12.978	0.6439	<0.01

**Figure 2:**
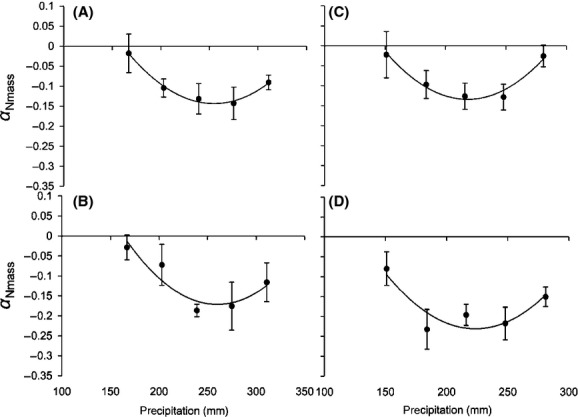
Relationship between *α*_Nmass_ under elevated CO_2_ (550 ppm) and precipitation. *S. baicalensis* (A), *S. bungeana* (B), *S. grandis* (C), and *S. breviflora* (D).

An elevated CO_2_ concentration led to a lower N_mass_ in the four *Stipa* species. However, the effect of elevated CO_2_ was closely related to the precipitation rate (Fig.1). The relative effect of elevated CO_2_ (550 ppm) on N_mass_ (*α*_Nmass_) showed a quadratic relationship with the precipitation level (Fig.2, Table3). This meant that the effect of elevated CO_2_ would be obvious within a particular precipitation range, but would disappear outside of this range. The effective precipitation ranges in which the N_mass_ of the four *Stipa* species responded to elevated CO_2_ (550 ppm) were calculated from the equations in Table3: *S. baicalensis* (163–343 mm), *S. bungeana* (164–355 mm), *S. grandis* (148–286 mm), and *S. breviflora* (130–316 mm). When the precipitation amount was 253, 260, 217, and 222 mm for *S. baicalensis*, *S. bungeana*, *S. grandis,* and *S. breviflora*, respectively, the effect of elevated CO_2_ (550 ppm) reached the maximum (Table3).

**Table 3:** Relationship between the effect of 550 ppm CO_2_ on *α*_Nmass_ and precipitation

Species	Equation	*R* ^*2*^	*P*	OP (mm)	ERP (mm)
*S. baicalensis*	*y* = 1.62E-05*x*^2^ − 0.0082*x *+ 0.9071	0.4314	0.0338	253	163–343
*S. bungeana*	*y* = 1.83E-05*x*^2^ − 0.0095*x *+ 1.0658	0.4130	0.0409	260	164–355
*S. grandis*	*y* = 2.58E-05*x*^2^ − 0.0112*x *+ 1.0935	0.4093	0.0425	217	148–286
*S. breviflora*	*y* = 2.58E-05*x*^2^ − 0.0115*x *+ 1.0574	0.4066	0.0437	222	130–316

OP, optimum precipitation represents the amount of precipitation when elevated CO_2_ had a maximal effect on N_mass_; ERP, effective range of precipitation shows the range of precipitation in which elevated CO_2_ affected N_mass_.

### Responses of leaf biomass and N_total_ to elevated CO_2_ and precipitation changes

**Figure 3:**
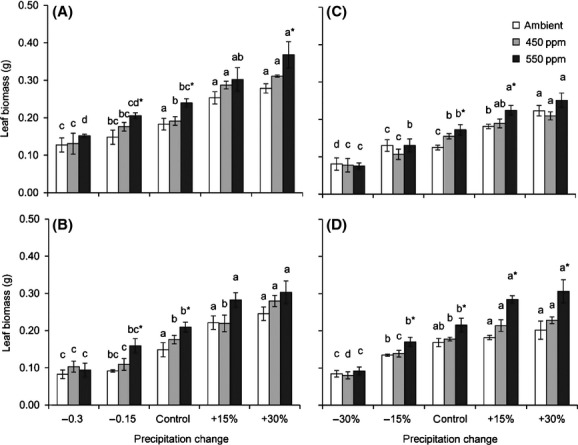
Interactive effects of changing precipitation and CO_2_ on leaf biomass of the four *Stipa* species: *S. baicalensis* (A), *S. bungeana* (B), *S. grandis* (C), and *S. breviflora* (D). See Fig.1 for notes.

Changes in precipitation significantly affected leaf biomass (Fig.3). Compared with the control, the leaf biomass of *S. baicalensis*, *S. bungeana*, *S. grandis,* and *S. breviflora* decreased 30.4%, 44.4%, 35.5%, and 49.8% (precipitation −30%) and increased 52.2%, 65.1%, 79.0%, and 19.8% (precipitation +30%), respectively, under ambient CO_2_ conditions. When the CO_2_ concentration elevated from ambient to 550 ppm, leaf biomass significantly increased. However, the effect of elevated CO_2_ on leaf biomass was also closely related to the precipitation rate, similar to N_mass_. Severe drought (precipitation −30%) restricted the effect of elevated CO_2_ concentration on leaf biomass (Fig.3).

**Figure 4:**
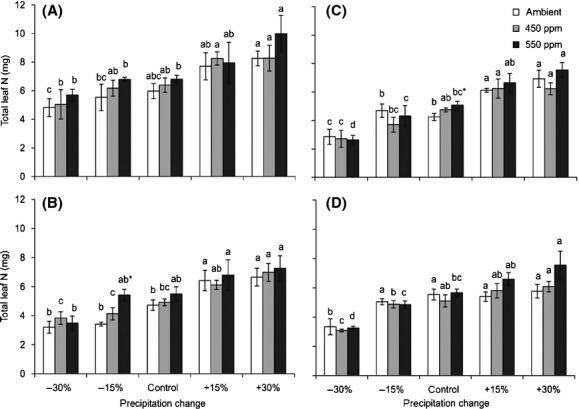
Interactive effects of changing precipitation and CO_2_ on the N_total_ of the four *Stipa* species: *S. baicalensis* (A), *S. bungeana* (B), *S. grandis* (C), and *S. breviflora* (D). See Fig.1 for notes.

Compared with the control, reduced precipitation increased the N_mass_ (Fig.1) but decreased the N_total_ of the four *Stipa* species (Fig.4). The N_total_ of *S. baicalensis*, *S. bungeana, S. grandis,* and *S. breviflora* decreased 19.3%, 32.3%, 32.6%, and 40.0% (precipitation −30%), respectively, under ambient CO_2_ conditions compared with the control. Although elevated CO_2_ increased the N_total_ of the four *Stipa* species, the effect was not significant expect under the −15% (*S. bungeana*) and control (*S. grandis*) precipitation conditions.

### Impacts of elevated CO_2_, precipitation changes and their interactions on N_mass_, leaf biomass and N_total_

Precipitation changes generally resulted in significant effects on the N_mass_, leaf biomass, and N_total_ of the four *Stipa* species (*P* < 0.001). The N_mass_ and leaf biomass changed with elevated CO_2_ concentration, but N_total_ was not significant. The interaction between elevated CO_2_ and precipitation changes had no significant effect on the N_mass_, leaf biomass, and N_total_ of the four *Stipa* species except for the leaf biomass of *S. breviflora* (Table4).

**Table 4:** F-values and significance levels (^*^P < 0.05; ^*^^*^P < 0.01; ^*^^*^^*^P < 0.001) from two-way ANOVAs for the main effects of CO_2_, precipitation and their interactions on N_mass_, leaf biomass and N_total_

	CO_2_		Precipitation		CO_2_ × Precipitation	
	*F*-values	df	*F*-values	df	*F*-values	df
*S. baicalensis*						
N_mass_	4.877^*^^*^^*^	2	21.98^*^^*^^*^	4	0.584	8
Biomass	11.80^*^^*^^*^	2	50.91^*^^*^^*^	4	0.534	8
N_total_	2.001	2	10.47^*^^*^^*^	4	0.240	8
*S. bungeana*						
N_mass_	7.077^*^^*^	2	43.66^*^^*^^*^	4	0.847	8
Biomass	10.70^*^^*^^*^	2	57.15^*^^*^^*^	4	0.775	8
N_total_	2.696	2	20.39^*^^*^^*^	4	0.551	8
*S. grandis*						
N_mass_	7.648^*^^*^	2	11.42^*^^*^^*^	4	0.877	8
Biomass	4.810^*^	2	58.73^*^^*^^*^	4	1.190	8
N_total_	1.376	2	34.83^*^^*^^*^	4	0.635	8
*S. breviflora*						
N_mass_	12.10^*^^*^^*^	2	20.70^*^^*^^*^	4	0.495	8
Biomass	23.58^*^^*^^*^	2	60.24^*^^*^^*^	4	2.295^*^	8
N_total_	3.056	2	25.90^*^^*^^*^	4	1.147	8

## Discussion

### Impacts of elevated CO_2_ and precipitation changes and their interactions on N_mass_

Nitrogen serves as one of the major mineral elements affecting plant growth, and leaves are the largest N sinks in plants. Leaf N_mass_ is closely related not only to the photosynthetic capacity of grass species (Sicher and Bunce 1997; Gerdol et al. 2000; Long et al. 2006; Duval et al. 2012) but also to the forage quality (Vitousek et al. 2002; Pleijel and Uddling 2012). This study confirmed earlier results that showed elevated CO_2_ concentrations decreased the N_mass_ of *Stipa* plants compared with those growing under ambient CO_2_ conditions (Ellsworth et al. 2004; Ainsworth and Long 2005; Crous et al. 2010; Lee et al. 2011). A reduction in N_mass_ is unfavorable for photosynthesis because it leads to a photosynthetic adaption phenomenon (Taub and Wang 2008; Lei et al. 2011) and is unfavorable for forage quality, which would cause a problem in the nutrition of animals.

Compared with the control precipitation rate used in this study, drought increased the N_mass_ of *Stipa*, which is consistent with previous reports (Luo et al. 2005; Huang et al. 2009). However, increased precipitation had no notable effect on N_mass_. The relationship between the N_mass_ of the four *Stipa* species and precipitation well fit a logarithmic function. N_mass_ gradually decreased with an increase in precipitation and was close to a constant value. A possible explanation for this phenomenon is that an element such as N must reach a certain concentration to allow plants to maintain their normal physiological activities. The increase in N_mass_ might enhance the number and activity of photosynthetic enzymes and improve the photosynthetic rate when plants are grown under drought conditions (Wright et al. 2001; Knight and Ackerly 2003; Huang et al. 2009). In addition, a higher N_mass_ level could increase intracellular osmotic pressure, which would strengthen the ability of plants to survive during drought, improve their water use efficiency and alleviate water-related stress (Wright et al. 2001; Huang et al. 2009; Novriyanti et al. 2012).

To date, there are limited reports on the interactive effect of changing precipitation and elevated CO_2_ on the N_mass_ of *Stipa*. This experiment showed that the elevated CO_2_ effect on leaf N_mass_ depended on the precipitation pattern. The changes in precipitation rates changed the sensitivity of N_mass_ to elevated CO_2_ concentrations. Precipitation is the most important factor in arid and semiarid ecosystems and plays a critical role in plant growth and physiological processes (Noy-Meir 1973; Morgan et al. 2004; Heisler-White et al. 2009). Precipitation limits the effect of elevated CO_2_ concentrations.

In this study, the patterns of leaf N_mass_ of the four *Stipa* species (*S. baicalensis*, *S. bungeana*, *S. grandis* and *S. breviflora*) in response to elevated CO_2_ and precipitation change were similar. However, elevated CO_2_ reduced N_mass_ in different precipitation ranges for the four *Stipa* species. The sensitivities of leaf N_mass_ of these four species to precipitation change were also different. The differential performance of the four *Stipa* species indicated that there may be species-specific leaf N_mass_ responses to precipitation change. This phenomenon might be related to the different biogeographic environments where the four *Stipa* species are distributed in nature. The leaf N_mass_ of *S. grandis* was the least sensitive to elevated CO_2_ and precipitation change among the four species. *S. grandis* is a principal species in typical steppe ecosystems (Zhang et al. 2007); it is more widely distributed than the other three species in the North China grassland in which *S. grandis* is better able to adapt to environmental change. Thus, *S. grandis* showed insensitivity to elevated CO_2_ and precipitation change in this experiment. *S. breviflora* thrives as a dominant species in desert steppe ecosystems (Zhang et al. 2007). This study showed that *S. breviflora* exposed to elevated CO_2_ was more sensitive than the other three species under drought conditions. *S. baicalensis* is as an important species in meadow steppe ecosystems in eastern Inner Mongolia (Zhang et al. 2007), which may explain why it was readily influenced by elevated CO_2_ under higher precipitation.

### Mechanisms of N_mass_ response to elevated CO_2_ and precipitation changes

**Figure 5:**
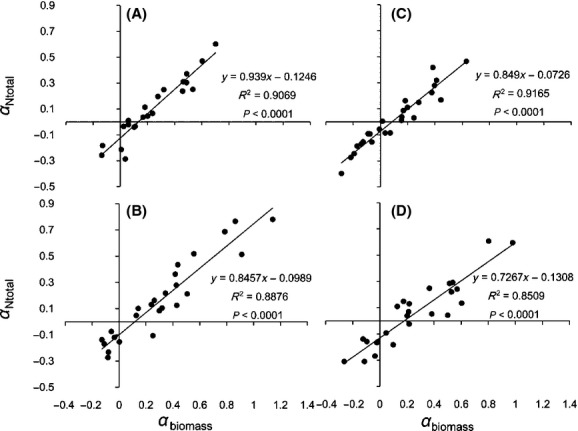
Relationship between *α*_Ntotal_ and *α*_biomass_ for *Stipa* under elevated CO_2_. *S. baicalensis* (A), *S. bungeana* (B), *S. grandis* (C), and *S. breviflora* (D).

Three hypotheses have been proposed in relation to the mechanisms by which N_mass_ responds to elevated CO_2_. (1) The growth dilution hypothesis: If the increase in the accumulation of leaf biomass is more than the increase in N acquisition under high CO_2_ concentration, N_mass_ will decrease (Yamakawa et al. 2004; Johnson 2006; Taub and Wang 2008; Duval et al. 2012). (2) The inhibition of N absorption and transport capacity hypothesis. Initially, elevated CO_2_ results in lower transpiration rates and increased water use efficiency; secondly, elevated CO_2_ affects the exudates of roots and changes soil pH, thus influencing N assimilation. Additionally, decreased N assimilation has also been explained as a result of an increase in N use efficiency and a decrease in N demand under elevated CO_2_ (Zerihun et al. 2000; Teng et al. 2006; Taub and Wang 2008; Bloom et al. 2010; Duval et al. 2012). (3) Both (1) and (2) coexist (Pleijel and Uddling 2012). Our results showed that although N_mass_ decreased, total leaf N (N_total_) increased under high CO_2_ concentration (Fig.3), which was consistent with previous results (Yin et al. 2011). We can test the mechanisms using the data of the relative effects of leaf biomass (*α*_biomass_) and total leaf N (*α*_Ntotal_). If the *α*_Ntotal_ data are plotted on the *y*-axis and the *α*_biomass_ data are plotted on the *x*-axis and the result is a linear regression with a slope between 0 and 1, this can be interpreted as a significant growth dilution effect. If a direct negative effect on N uptake exists that is unrelated to the effect on leaf biomass, in addition to the growth dilution effect, there will be a significant intercept on the *x*- and *y*-axes (Taub and Wang 2008; Pleijel and Uddling 2012). The relationship between *α*_Ntotal_ and *α*_biomass_ for *Stipa* under elevated CO_2_ showed that N_mass_ decreased because of the combined effect of growth dilution (the slope was between 0 and 1) and assimilation inhibition (the intercept on the *y*-axis was smaller than 0) (Fig.5), which is the same as the results of previous studies (Teng et al. 2006; Taub and Wang 2008; Pleijel and Uddling 2012). The slope (*k*) and *y*-axis intercept (¦*b*¦) reflect the respective degree to which the growth dilution and assimilation capacity affect N_mass_. The sensitivity of the four *Stipa* species can be listed as: *S. breviflora *> *S. bungeana *> *S. grandis *> *S. baicalensis* for growth dilution, and *S. breviflora *> *S. baicalensis *> *S. bungeana > S. grandis* for decreased N assimilation capacity.

**Figure 6:**
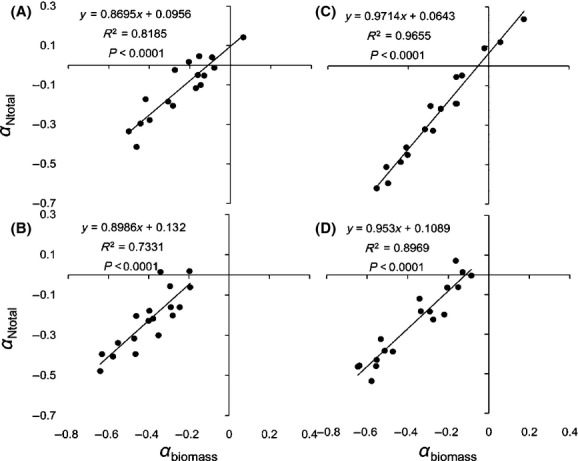
Relationship between *α*_Ntotal_ and *α*_biomass_ for *Stipa* under reduced precipitation. *S. baicalensis* (A), *S. bungeana* (B), *S. grandis* (C), and *S. breviflora* (D).

Compared with the control, reduced precipitation increased the N_mass_ but decreased the N_total_ of *Stipa* L. (Fig.3). Based on the relationship between *α*_Ntotal_ (as *y*-axis) and *α*_biomass_ (as *x*-axis) of *Stipa* (Fig.6), the increase in N_mass_ under drought can be explained in two ways. First, the decrease in leaf biomass accumulation was larger than the decrease in N_total_ accumulation. Second, drought strengthened N uptake and transport (the intercept on the *y*-axis was >0). It is possible that N_mass_ increased because more N was needed to maintain a high osmotic pressure or because drought increased the root–shoot ratio and more roots transported N to the same volume of leaves (Jiang et al. 2004; Pan et al. 2008; Duval et al. 2012).

## Conclusions

In this experiment, we studied the interactive effects of CO_2_ concentration (ambient, 450 and 550 ppm) and precipitation (−30%, −15%, control, +15%, and +30% based on average monthly precipitation from 1978 to 2007 in the provinces that support the populations of the four species) on leaf N of four species: *S. baicalensis S. bungeana*, *S. grandis,* and *S. breviflora*. The results suggested the following: (1) Elevated CO_2_ decreased the N_mass_ but increased the N_total_ of *Stipa* L. The decrease in N_mass_ was caused by the combination of growth dilution and assimilation inhibition. The effect of elevated CO_2_ was influenced by precipitation: Within a precipitation range, the effect was obvious; however, the effect disappeared outside of that range. (2) Compared with the control precipitation, reduced precipitation increased the N_mass_ of the four *Stipa* species, but increased precipitation had no significant effect on N_mass_. The increase in N_mass_ under drought conditions might have resulted from two causes: The decrease in leaf biomass accumulation was greater than the decrease in N_total_ accumulation, and drought strengthens N uptake and transport. The relationship between the N_mass_ of the four *Stipa* species and precipitation was described using a logarithmic function. Elevated CO_2_ exacerbated the effect of precipitation on N_mass_. (3) The sensitivity of the N_mass_ of the four species to precipitation was ranked as: *S. bungeana *> *S. breviflora > S. baicalensis > S. grandis*. The N_mass_ of *S. grandis* was the least sensitive among these four species. Under drought conditions, the effects of elevated CO_2_ on *S. breviflora* were the most obvious among the four species.
